# Adverse Effects of Proton Pump Inhibitors on Platelet Count: A Case Report and Review of the Literature

**DOI:** 10.1155/2018/4294805

**Published:** 2018-04-30

**Authors:** Subhajit Mukherjee, Tanima Jana, Jen-Jung Pan

**Affiliations:** ^1^Division of Gastroenterology, Hepatology and Nutrition, Department of Internal Medicine, The University of Texas Health Science Center, Houston, TX 77030, USA; ^2^Division of Gastroenterology and Hepatology, Department of Medicine, University of Arizona College of Medicine, Tucson, AZ 85724, USA

## Abstract

Proton pump inhibitors (PPIs) are the most effective and preferred class of drugs used to treat peptic ulcer disease, gastroesophageal reflux disease, and other diseases associated with increased production of gastric acid. PPIs in general have an excellent long-term safety profile and are well-tolerated. However, studies have shown some adverse reactions (e.g., osteoporosis,* Clostridium difficile*-associated diarrhea, Vitamin B12 and iron deficiency, and acute interstitial nephritis) on long-term PPI use. Thrombocytopenia attributed to use of PPIs has been described in a few case reports and a retrospective study. In this case report, we describe a case of PPI-induced thrombocytopenia. In our patient, thrombocytopenia immediately developed after the initiation of PPI on two separate occasions and resolved after its discontinuation. The strong association found in our case implies the potential role of PPI in causing this rare but serious adverse reaction. Based on this case report and the observation from other studies, a PPI-induced adverse event should be considered as a possible etiology for new-onset idiopathic thrombocytopenia.

## 1. Introduction

Proton pump inhibitors (PPIs) are the most commonly used class of drugs for the treatment of gastric acid-related disorders [1]. PPIs inhibit gastric acid production by inhibiting the gastric parietal cell hydrogen potassium ATPase, which is needed for the final step of acid secretion [1, 2]. PPIs are the most potent inhibitor of this enzyme currently available and hence their therapeutic role in the treatment of acid-related disorders is well-established [1, 2]. Conditions in which PPIs are more effective and commonly used include peptic ulcer disease, gastroesophageal reflux disease (GERD), Zollinger-Ellison syndrome, eradication of* Helicobacter pylori* infection, treatment of bleeding gastroduodenal ulcers and esophageal strictures, and the maintenance therapy for Barrett's esophagus [3–12].

Omeprazole was the first PPI used in clinical practice (in the late 1980s). Since then, several other PPIs have become available [13]. Although the different types of PPIs differ with regard to their pharmacokinetic profile, bioavailability, and route of excretion, their clinical effectiveness is very similar [1, 14]. In fact, findings from various clinical trials have been unsuccessful in selecting one type of PPI over another based on their therapeutic efficacy and cost-effectiveness [14, 15]. PPIs in general are very safe drugs, especially if they are used for short-term purposes, but recent literature has shown concern with their long-term use [16]. Some of the adverse sequelae of long-term PPI use include osteoporosis with increased risk of bone fractures,* Clostridium difficile*-associated diarrhea, pneumonia, hypomagnesemia, Vitamin B12 deficiency, iron deficiency, acute interstitial nephritis, hypergastrinemia, and chronic atrophic gastritis [17–25]. The Food and Drug Administration (FDA) has raised safety concerns regarding long-term PPI use mainly for osteoporosis,* Clostridium difficile*-associated diarrhea, and hypomagnesemia. However, in their 2008 guidelines for GERD management, the American Gastroenterological Association (AGA) did not recommend any routine safety monitoring in patients on long-term PPI, due to insufficient evidence for these adverse events [16]. In fact, none of the gastroenterology societies have recommended any surveillance for potential adverse risks in long-term PPI users.

Short-term adverse events of PPIs are rare. Only a few case reports have shown an increased incidence of rebound gastrointestinal symptoms and community-acquired pneumonia after short-term PPI use [26, 27]. While nine case reports and one retrospective study have demonstrated that PPIs may cause thrombocytopenia, another large retrospective study failed to show any increased incidence of thrombocytopenia after PPI use [28–38].

In this report, we describe a case of life-threatening thrombocytopenia after PPI use. In our patient, the thrombocytopenia occurred after the initiation of PPI and resolved after its discontinuation. The causality of PPI with regard to thrombocytopenia in this particular case was further strengthened by the observation of another episode of thrombocytopenia when the PPI was reintroduced. Complete recovery of the platelet count only occurred when the PPI was stopped and, hence, the PPI was subsequently listed as a drug allergy for this patient.

## 2. Case

A 35-year-old Hispanic female was admitted for worsening upper abdominal pain, nausea, and vomiting. She had a past medical history of heartburn which was being treated with PPI. She was initially seen in the emergency department for worsening epigastric abdominal pain and was discharged home on daily omeprazole. She returned to her primary care clinic 2 months later complaining of similar symptoms while being on omeprazole. Since omeprazole was not effective, she was switched to esomeprazole. Two months later, she visited her home country of El Salvador and was evaluated for abdominal pain. Due to her persistent symptoms, cholecystectomy was performed, without much relief of her symptoms. Patient reported that she was unable to take the initially prescribed esomeprazole secondary to financial issues and was not on any acid-suppressing medications in the previous 2 months. Upper endoscopy was then performed and it showed multiple gastric ulcers. She was then started on pantoprazole. After returning to the United States, she continued to have pain and started taking nonsteroidal anti-inflammatory drugs (NSAIDs) for relief. Subsequently, she visited her primary care office with worsening of her pain associated with nausea and vomiting. During this clinic visit, she was switched to dexlansoprazole and was asked to come to the emergency department if she continued to have symptoms. She returned to the emergency department the next day for further evaluation of her worsening symptoms. On initial evaluation in the emergency department, she was afebrile and had stable hemodynamics. She endorsed severe abdominal pain and a 30-pound weight loss over the last year, but denied any hematemesis, melena, or hematochezia. Laboratory evaluation revealed white blood cell count of 26.5 × 10^3^/mm^3^, hemoglobin 13.8 g/dl, and platelet count of 116 × 10^3^/mm^3^. On review of laboratory data, patient's last platelet count checked 6 months priorly was normal (264 × 10^3^/mm^3^) and had been obtained before the patient was started on a PPI for the first time. No other laboratory tests had been obtained until this recent emergency department visit. Therefore, the effect of PPI on the platelet count for the next 6 months after initiation of therapy was not available to us. Chemistry panel, liver function, urinalysis, and blood/urine cultures were negative. CT imaging of the abdomen and pelvis showed diffuse steatosis but was otherwise normal.

Gastroenterology was consulted and due to refractory abdominal pain, weight loss, and NSAID use, an upper endoscope was recommended. Additionally, intravenous esomeprazole twice daily was started. Her platelet count continued to drop, falling to 72 × 10^3^/mm^3^ the next day and to 12 × 10^3^/mm^3^ the day after. Hematology was consulted for the rapid drop in platelet count and the etiology was thought to be secondary to drug-induced thrombocytopenia, infection, or idiopathic thrombocytopenic purpura. Of note, the patient did not have any history of bleeding or clotting disorders. Additionally, there was no evidence of hemolysis on the peripheral blood smear and the patient was not coagulopathic. On review of medications, since there were no other drugs (except for one prophylactic dose of heparin) that could be attributed to thrombocytopenia, it was recommended to hold the PPI. The PPI was then stopped, and platelet count recovered to 99 × 10^3^/mm^3^ within two days. Upper endoscope performed at that time revealed nonspecific gastritis. Biopsies were found to be negative for* Helicobacter pylori* infection. Due to the spontaneously improved platelet count, antibodies to heparin-platelet factor 4 complex were not checked to rule out heparin-induced thrombocytopenia. Since our patient's platelet count normalized after stopping PPI, this current episode of thrombocytopenia was deemed likely secondary to PPI use.

**Figure 1:**
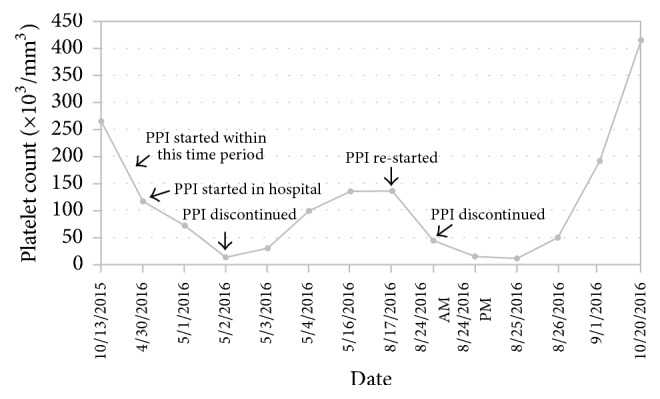
Platelet count trend of the patient. Thrombocytopenia developed after starting PPI for the first time and later on when it was restarted. Platelet count recovered after PPI was discontinued on both occasions (*x*-axis: actual date; *y*-axis: platelet count in 10^3^/mm^3^).

Our patient was subsequently discharged home, but continued to have persistent epigastric pain. She tried a H2 (histamine 2) receptor antagonist with minimal symptom relief. She was next seen in the Gastroenterology Clinic. At this time, the platelet count was 135 × 10^3^/mm^3^. During this visit, the question of whether patient's thrombocytopenia was truly related to PPI use was revisited, given that heparin-induced thrombocytopenia was not ruled out. Since a PPI was warranted due to her persistent symptoms, the decision was made to restart dexlansoprazole with close follow-up. She ultimately got readmitted to the hospital 7 days later for persistent epigastric pain (while on PPI). This time the platelet count was found to have decreased further to 43 × 10^3^/mm^3^. The platelet count continued to drop, similar to her prior admission while on PPI, with the lowest count being 10 × 10^3^/mm^3^. PPI was held because of prior concern for PPI-induced thrombocytopenia. On this admission, our patient did not receive any heparin products and peripheral blood smear was not consistent with hemolysis. She did not receive any medications known to cause thrombocytopenia. Platelet count improved to 50 × 10^3^/mm^3^ while off PPI and she was discharged home. Patient's symptoms improved on H2 antagonist, sucralfate, and pain control with morphine. On this admission, PPIs were listed as a drug allergy and documented in the patient's medical record. She was seen in the Gastroenterology Clinic after hospital discharge and it was noted that her symptoms were partially controlled on H2 antagonist, sucralfate, and scopolamine (which she received from her home country for control of nausea). Platelet count ultimately improved to 415 × 10^3^/mm^3^. A complete pictorial description of our patient's platelet count is shown in Figure 1.

## 3. Discussion

PPIs are an important class of medications for long-term use in patients suffering from GERD and Barrett's esophagus [8, 11]. They are also important for the treatment of peptic ulcers, especially in acute settings when patients present with upper gastrointestinal bleeding [12]. Generally, PPIs are very safe medications, but a few case reports have implicated their role in causing thrombocytopenia [28, 33]. This side effect of PPIs should be taken seriously, as the drop in the platelet count in our patient was very severe, and this side effect poses significant consequences such as life-threatening bleeding.

Drug-induced thrombocytopenia is a diagnosis of exclusion and its incidence is less than 1% in the general population for nonheparin drug products [39]. In our patient, the temporal relationship of the drop in platelet count with the introduction of PPI, along with the subsequent revival of the count upon its withdrawal, was sufficient to implicate PPI use as the reason for the thrombocytopenia. A reasonable first step in the evaluation and diagnosis of drug-induced thrombocytopenia is to discontinue the offending drug and look for the normalization of platelet counts. However,* in vitro* testing for the detection of drug-dependent antibodies provides a direct analytical method to diagnose this condition. This test can be used as an adjunct to clinical findings in making a diagnosis of drug-induced thrombocytopenia and also for drug surveillance [40].

Previous case reports have shown similar effects of PPI-induced thrombocytopenia for several types of PPI (e.g., pantoprazole, lansoprazole, and omeprazole). In all cases, platelet count dropped the very next day after starting PPI. The first case of pantoprazole-induced thrombocytopenia was reported by Watson et al. [28]. Subsequently, other authors have also documented a similar effect after pantoprazole administration [29–31, 34]. In keeping with these observations of pantoprazole-induced thrombocytopenia, Binnetoğlu et al. in a retrospective study of 35 patients demonstrated significant thrombocytopenia after pantoprazole infusion [33]. However, Dotan et al., in a retrospective study of 468 hospitalized patients, failed to demonstrate increased incidence of thrombocytopenia after pantoprazole use [32].

A small number of case reports have demonstrated thrombocytopenia with different types of PPI. While Zlabek and Anderson and Ogoshi et al. documented evidence of thrombocytopenia after oral intake of lansoprazole, Hayashibara and Rudelli et al. described similar effects of thrombocytopenia with omeprazole use [35–38]. Finally, Ranzino et al. reported a case of thrombocytopenia after coadministration of esomeprazole and hydantoin, but a direct causal relationship of esomeprazole use and thrombocytopenia was not established in their study [41].

In our case, both esomeprazole and dexlansoprazole caused thrombocytopenia, so it is likely that this effect is not drug-specific, but is rather a class effect. Additionally, both intravenous and oral use of PPI caused similar effects in dropping platelet counts, with platelet counts decreasing as low as 10 × 10^3^/mm^3^. Hematology work-up including examination of a peripheral smear was also done, excluding other causes of thrombocytopenia.

Based on the findings from these case reports and from our observation, it appears that PPIs can cause thrombocytopenia. However, the mechanism of action is unknown. Future research should be focused on discovering how this class of drugs, which primarily works by inhibiting hydrogen potassium ATPase, can cause thrombocytopenia. Irrespective of the mechanism, we should exercise caution when prescribing PPIs for long-term use and monitor patient's platelet counts carefully while on PPIs.
